# Barriers and facilitators to the implementation of a national multisectoral action plan for the prevention and control of noncommunicable diseases in Nepal: perspectives of stakeholders

**DOI:** 10.1080/16549716.2021.1963069

**Published:** 2021-08-27

**Authors:** Meghnath Dhimal, Mandira Lamichhane Dhimal, Sushma Dahal, Mahendra Prasad Shrestha, Pradip Gyanwali, Ruitai Shao, Bente Mikkelsen, Kremlin Wickramasinghe, Robert Marten, Anjani Kumar Jha, Nick Townsend

**Affiliations:** a Nepal Health Research Council (NHRC) Government of Nepal Ministry of Health and Population Complex, Kathmandu, Nepal; b Policy Research Institute, Kathmandu, Nepal; c Department of Health Sciences, Ministry of Health and Population, Kathmandu, Nepal; d Department of Noncommunicable Diseases, World Health Organization, Headquarters, Geneva, Switzerland; e Division of Non-communicable Diseases and Promoting Health through the Life-course, WHO Regional Office for Europe, Copenhagen, Denmark; f WHO European Office for Prevention and Control of NCDs, Moscow, Russia; g Alliance for Health Policy and Systems Research, WHO, Geneva, Switzerland; h Department for Health, University of Bath, Bath, UK

**Keywords:** Multisectoral, noncommunicable disease, prevention, policy, implementation

## Abstract

**Background:**

Nepal adopted the Multisectoral Action Plan for the Prevention and Control of Non-Communicable Diseases (MSAP) in 2014. Implementation of the plan has been challenging, with limited participation from non-health sectors.

**Objectives:**

The overall aim of the study was to gain the perspectives of key stakeholders involved in the Nepal MSAP on the barriers and facilitators to its implementation, through the participation of relevant sectors in the plan.

**Methods:**

We held face-to-face semi-structured interviews with 12 stakeholders working in sectors involved in the MSAP. These sectors included the Office of the Prime Minister and Council of Ministries; Ministry of Health and Population (MOHP); Ministry of Education, Science and Technology; Ministry of Forest and Environment; academia; and professional organizations. Thematic analysis of transcripts was used to identify themes on awareness of NCDs, awareness of the MSAP, and barriers and facilitators to participation in the MSAP.

**Results:**

Participants recognised NCDs as a growing and major burden in Nepal. However, a number of participants were not familiar with the MSAP, identifying a lack of leadership and poor dissemination. Political and systemic transformation, since the adoption of the MSAP, was seen as a key barrier to implementation. International commitments to develop multisectoral action made by the Government of Nepal were identified as drivers. The recent establishment of a separate section for NCDs and Mental Health within the Department of Health Services of MOHP and the promotion of a Health in All Policies (HiAP) approach in recent national documents, were both considered to support implementation.

**Conclusions:**

The establishment of permanent multisectoral or multistakeholder mechanisms has been challenging despite strong political calls for their development. Moving beyond 2020, multisectoral action plans should engage with stakeholders from federal, provincial and local governments in order to develop costed action plans with specific roles and responsibilities for each sector.

## Background

The prevention and control of noncommunicable disease (NCDs) has been prioritised in the international agenda, recognising the growing burden that NCDs represent globally. In 2011 the United Nations (UN) General Assembly first High Level Meeting on the Prevention and Control of NCDs received commitments from national leaders to take measures to tackle NCDs[1]. Since then commitments have been adopted at two further UN high-level meetings, within the 2030 Agenda for Sustainable Development and through the World Health Organization (WHO) governing body’s resolutions and decisions. Culminating in the integration of NCDs within the Sustainable Development Goals (SDG); in particular SDG 3.4, which targets reducing premature mortality from NCDs by one-third by 2030 [2].

Within the second High Level Meeting of the UN General Assembly in 2014, Member States agreed to four time-bound commitments. The second of which, ‘Consider developing or strengthening national multisectoral policies and plans’ recognises that many of the drivers of NCDs and their risk factors lie outside the responsibility of the national health sectors [3]. Such that a multisectoral or multistakeholder approach, often termed ‘health-in-all-policies’, ‘whole-of-government’, ‘whole-of society’, or ‘cross-sectoral’ [4,5], is needed to address NCDs risk factors and determinants in an effective way [6,7].

The establishment of permanent multisectoral or multistakeholder mechanisms has been problematic, despite strong political calls for their implementation. With the challenges to multisectoral action thought to be more acute in low-income and middle-income countries (LMICs) where institutions are frequently weak, and fragmentated, even within the health sector, can undermine coordination [8]. The 2018 Political Declaration for the High-Level Meeting on NCDs called on Heads of State and governments to strengthen commitment in this area, by providing strategic leadership, coordinated action and response for the prevention and control of NCDs [9]. This was timely as the 2030 Sustainable Development Agenda challenges countries to move towards whole-of government and whole-of-society approaches that ‘leave no one behind’ [9,10].

The UN SDGs provide a renewed impetus for joined-up action to address complex, contemporary problems and for the achievement of health and good governance. The SDG goals are ‘integrated and indivisible’ and require multistakeholder and multisectoral action to achieve them [11]. In particular SDG 17, which calls for cooperation, collaboration and partnership between government, civil society and businesses and encourages ‘the use of multistakeholder partnerships’ but also SDG 3 which aims to ‘ensure healthy lives and promote wellbeing for all at all ages’ [2,12,13].

It is concerning, therefore, that WHO Country Capacity Surveys found that less than half of reporting countries have an operational national multi-sectoral commission, agency or mechanism [14]. Leading to recommendations from the WHO Independent High-level Commission on NCDs to place an emphasis on implementation at a country level and to promote exchange of experiences of countries in implementing such mechanisms [14]. Supporting calls for the development of a implementation research agenda in the governance of multisectoral action, which could provide a rallying point for a community of learning and practice [15]. Using real-world experiences of how multisectoral collaboration works [16] to support knowledge management and convene peer learning [17].

Country Capacity Surveys reported great variation between regions in the proportion of Member States with operational MSAPs. The South-East Asia Region (WHO SEAR) demonstrated the greatest coverage, with 10 out of 11 Member States (91%) reporting operational NCD National Coordination Mechanisms (NCMs). This compared to the next highest of 57% in the WHO European Region (WHO EUR) and a low of 19% in the WHO African Region (WHO AFR)(14). A 2018 situational analysis of multisectoral NCD governance mechanisms within South East Asia, commissioned by the WHO South-East Asian Regional Office (WHO SEARO), found that all countries in the region had adopted an MSAP to address NCDs. However, they reported that frequency of meetings was less than had been envisaged and that subnational NCD responses was largely limited to the health sector, with functional NCD coordination mechanisms yet to emerge at lower levels. The lack of adequate human and financial resources were among the main barriers to NCD governance and multisectoral response in all SEAR countries. With the report recommending facilitation of peer-to-peer learning, and the documentation and dissemination of country experiences and good practice(18).

This study presents findings from interviews from stakeholders involved in the Multisectoral Action Plan for the Prevention and Control of Non Communicable Diseases (2014–2020) for Nepal (MSAP) [18], a low-income country with a population of around 28 million people [19], in which NCDs account for 66% of total deaths [20]. Morbidity and mortality rates due to NCDs have more than doubled over the past 25 years in Nepal, with approximately half of the burden of NCDs occurring under the age of 40 [21]. The rising burden of NCDs in the country has been attributed to unplanned urbanization, changes in lifestyle, demographic and economic transitions, along with globalization, leading to increased behavioural risk factors including tobacco use, alcohol consumption, unhealthy diets and physical inactivity [22]. With data from WHO STEPwise Approach to NCD Risk Factor Surveillance (STEPS) surveys finding no significant improvement in the prevalence of these risk factors in Nepal between 2013 and 2019 [23].

In order to address the increasing burden of NCDs in Nepal, the Government of Nepal’s Ministry of Health and Population (MOHP), formulated the MSAP with support from the WHO. Within the MSAP key health sector and non-sector synergies were identified, with the MOHP assuming overall coordination and leadership for implementing the action plan under the guidance of the national steering committee. The MSAP, anchored on the active participation of various sectors of the Government in a whole-of-government and whole-of-society approach, was discussed extensively with key line ministries and stakeholders and was endorsed by the Government of Nepal [18]. The overarching goal of the MSAP was to reduce preventable morbidity, avoidable disability and premature mortality due to NCDs in Nepal, articulated through 10 targets to be achieved by 2025. These targets include the nine voluntary global targets described in the Global Action Plan for the Prevention and Control of NCDs 2013–2020 [3], with an additional one of a 50% relative reduction in the proportion of households using solid fuels as the primary source of cooking, included to align with SEAR Regional NCD targets [18].

To date, implementation of the MSAP has been challenging. The 2018 WHO SEARO review found that within Nepal good practice included distinct technical support, champion leadership and the leveraging infrastructure of non-health sectors. However, it also reported that the NCD governance committees had only met twice, with the first meeting attended by half its member agencies. Delays in meetings were said to be due to the ongoing nationwide political processes of elections and restructuring of ministries, which had delayed the functioning of the country’s NCD governance committees. With most stakeholder ministries ‘busy’ restructuring their agencies and realigning their provincial approaches(18).

The WHO SEAR Review involved a desk review of country documents pertaining to governance mechanisms for NCDs, and policies and programmes resulting from multisectoral action, along with interviews of one NCD focal point in each country. It was not, therefore, able to gain the perspectives of a range of health and non-health stakeholders involved in the MSAPs [24].

The overall objective of this study is to document the perspectives of key stakeholders involved in the Nepal MSAP on the barriers and facilitators to the participation of relevant sectors in the plan and its overall implementation. This work is timely as the Government of Nepal is currently formulating a second MSAP for the years 2021–2025. Assessment of facilitating factors and barriers to implementation of the preceding MSAP (2014–2020), in the country, should be useful in supporting implementation of the follow-up programme.

## Methods

We interviewed a total of 12 individuals for this study, from sectors and organisations involved in the MSAP (Table 1). Participants were identified through purposive sampling, as they were responsible for the implementation of the MSAP within their organization. Participants were proposed by ministries identified from the MSAP, there were no non-responses from those invited and all agreed to take part.

**Table 1.** Participants by organisation and role

Participant Number	Organisation
1	Department of Environment
2	Office of Prime Minister
3	National Health Training Centre
4	Patan Academy of Health Sciences
5	Ministry of Health and Population (MOHP)
6	District Public Health Office, Laitpur
7	Health Coordination Division, MOHP
8	Ministry of Education, Science and Technology (MOSTE)
9	NCD and Mental health section, Epidemiology and Disease Control Division, Department of Health Services
10	Policy, Planning and Coordination Division, MOHP
11	Provincial Health Director
12	Ministry of Agriculture and Cooperatives

All interviews were carried out by the first author (MD), an experienced qualitative researcher with a background in NCD research in Nepal. Interviews were conducted in English or Nepali, or both, as per the request of the participants, between 10th November and 20 December 2018. Interviews were recorded using smart mobile phones, with the informed consent of participants. Upon request of participants, four interviews were not recorded and notes were prepared.

All interviews were semi-structured but followed an interview schedule investigating the perceptions of participants on multisectoral action on NCDs, familiarity with and participation in the MSAP, as well as barriers and facilitators to its implementation. The semi-structured nature of the interviews allowed participants to introduce topics and themes not covered in the schedule.

The interviews were transcribed verbatim and those carried out in Nepali translated into English. In addition, notes were taken and summarized after each interview. We used a combination of deductive and inductive thematic analysis to identify themes on these topics. The initial identification of codes was inductive, following key questions. These questions were used as broad codes, within which a more deductive approach was used, as participants identified themes related to them. Coding was carried out by two researchers with support from the first author (MD), cross checking was carried out during the coding stage to ensure consistency between coders.

## Results

Themes were identified within three broad codes: (1) Awareness of NCDs and MSAP, (2) Barriers to implementation of, and participation in, MSAP, and (3) Facilitators to implementation of, and participation in, MSAP

## Awareness of NCDs and MSAP

### Awareness of NCDs

All participants identified NCDs as a growing and major burden in Nepal. Many discussed personal experiences of NCDs through families and friends, although surveillance data were identified as the major factor in supporting policy action, with the WHO STEPS survey seen as important in this.

We are gradually understanding the magnitude of problem of NCDs … . (the) burden of NCDs is rapidly increasing in Nepal and in each family at least one person has risk factor of NCDs or suffer from NCDs. Not only from my personal experience, our national studies on NCDs such as NCD STEPS survey show increasing risk factors of NCDs … Such situation has compelled us to take response through formulation of policies and plans in Nepal.-P7

However, it was reported that there had been a delay in acknowledging NCDs as a problem and in recognising the role sectors and institutions can play in prevention and control.

For NCD prevention and control, the important parts are what is the role of the individual, family and institutional wise? … We are in late from the part of NCD in institutional level because of reality that there was a lack of awareness about NCDs. We, policy maker and government sector, were given the priority of NCD in a delayed process … . prevalence was high … . From the part of government and policy there was delay in planning.–P3

## Sectors role in NCD prevention and control

NCD prevention and control was seen to be tackled through a number of policy documents and committees. The majority of participants reported a high level of participation from their sector in at least one NCD prevention and control committee, including those at a national, regional or district level. Although they also agreed that the responsibility for the prevention and control of NCDs lay with the MOHP and that a lack of budget or funding to support NCD-focused action, within their sector, hindered their involvement.

We have agriculture policy which talk about food safety and security but do not have specific policy for NCDs. Health Ministry is responsible for formulating such policy and we do not have any specific budget for NCDs.-P12

Those from sectors outside of health reported that they did not have specific policies targeted at NCD prevention and control. However, there was acknowledgement that the responsibility of these sectors included determinants of NCDs, such that although they were not targeting NCDs specifically, they had policies which may impact on them.

We do not have specific policies to address NCDs. However, as per our commitment in multilateral environmental agreements, we have development environmental acts, regulations and policies. One relevant work for NCDs could be, I think, our work on development of air pollution management action plan … we have also developed and implemented national standards of air quality for both indoor and ambient air.–P1

## Familiarity with and relevance of MSAP

Acknowledgment that many of the determinants of NCDs lay outside of MOPH responsibilities, was said to encourage participants to support the call for multisectoral action on the prevention and control of NCDs.

I want to stress that multisectoral collaboration is key for combating NCDs as health sector alone cannot deal with it –P5

However, a number of participants were not familiar with the MSAP, with those who had heard of it reporting that they were not well informed on its role or action. This was particularly the case for those who had only recently moved into their position, with information on the MSAP proving slow to be transferred.

I am not much familiar about this plan, but food sector is of course important for NCDs … As I am new, nobody has handover any information or document about this. I will see in documents. Frankly speaking, I am not aware about this national document.–P12

In line with this, a number of interviewees felt that there was little participation from their sector within the MSAP, although individuals were sometimes invited to input.

I don’t think there is provision of academic sector participation on different committees of NCDs prevention and control within the framework of MSAP. However, I sometimes participate in the discussion meeting when invited by governmental and non-governmental organizations.–P4

Those who worked at a provincial level reported that national leadership within their sector did participate, with multisectoral action perceived as national level action rather than provincial, despite a focus on NCDs at the local level.

The Ministry participates in high level and national committee for NCDs, but I do not know exactly who participates and how often meetings are organized. We have not set up such committee in provinces, though we are also working on NCDs–P11

Those who worked at a district level reported that they were not familiar with any specific policy or action plan on NCDs, although they acknowledged that many policy documents had been developed by the MOHP. They identified that a lack of costed action plan was a challenge to implementation, particularly due to the number of policy documents.

We develop many documents, but their implementation part is usually challenged as required resources are not ensured in such plans. Hence, I suggest to develop costed action plan … As NCDs are rapidly increasing in the community, we need more health promotion programmes and all three tiers of Governments i.e. local, provincial and central government should develop mechanisms for actions-P6

## Barriers to implementation of, and participation in, MSAP

### Restructuring of institutions in the federal context

The major barrier to implementation of the MSAP, and a reason for a lack of participation in it, identified by participants was a rapid political transformation, from central government to a federal structure, occurring since adoption of the MSAP. As a result, there was a transition period for restructuring the institutions at federal, provincial and local level.

We are struggling to define the health system structure at federal, provincial and local level. This has limited participation in MSAP as defined in the document.-P7

In order to improve participation in the MSAP, participants reported that it should be updated to align with the new federal structure.

First of all, we need to update MSAP to suit new federal structure of Nepal with clear role and responsibilities of local, provincial and federal governments.–P11

## Lack of committees at provincial level

This restructuring meant that formation of provincial- and district-level MSAP committees was challenging. Such that despite work being done at the national level to establish the MSAP, this had not been supported by the required work at the provincial level.

We do not have any direction from ministries to form NCD committees at regional and provincial levels, we do not have such committees.—P11

## Inadequate leadership

Participants suggested that they felt that there was a lack of clear and identified leadership within the MSAP. They agreed it should be led by the MOHP but acknowledged that the MOHP itself could not form provincial level committees. This meant that there was no focus point for sectors involved in the MSAP to coordinate with and that not enough had been done to drive the MSAP agenda.

Lack of institutional memory, wider dissemination, ownership, advocacy and leadership of the MOHP limited implementation of MSAP in Nepal … . We could not identify coordinating focal person within the MOHP and interact/advocate with line ministries for implementation of MSAP.-P7

## Poor dissemination and sharing of the MSAP

It was reported that the MSAP was disseminated through a workshop when it was first prepared in 2014. However, participants felt that there had been no regular dissemination and communication about the plan to new health officers of the MOHP and line ministries since then. Additionally, during staff transfer procedures there was poor organization of the handover of activities between incoming and outgoing officers.

In our Government system, [the] transfer of staff is very frequent and there is poor culture of handover. As a result, maintaining institutional memory and following up of past initiative is difficult.—P8

## No designated focal persons in line ministries

Frequent changes of personnel in all sectors resulted in a lack of focal point in each ministry and a lack of consistent relationships within the MSAP. As a result, it was reportedly difficult to coordinate and communicate between the sectors in order to work on NCD issues.

No designated unit or person in our ministry and frequent change of roles and responsibility of persons made it difficult to follow up progress.—P12

Within the MSAP the ‘Curative Division’ of the MOHP was given responsibility for implementation, but in the new systems structure there was no such division, leading to confusion with in the MOHP about the lead division/focal person. Participants suggested that each sector participating in the MSAP should have a focal point, either an individual or a committee, who would take responsibility for that sectors participation. Supported by the establishment of a multisectoral NCD commission.

In my opinion, Ministry of Health should take lead role and need to guide other sectors about how NCDs burden can be minimized. Each relevant Ministry should set up unit or sector for multisectoral coordination including for NCDs … . I think we should establish a multisectoral NCD Commission … –P2

## Limited resources

A lack of resources and funds to support multisectoral engagement, along with NCD developments, especially for sectors outside of health was identified as a limiting factor in working on them. This was in part due to the lack of national programmes for the prevention and control of NCDs, with limited resources for supporting programmes such as the WHO Package of Essential Noncommunicable Disease Interventions (WHO PEN).

Allocation of limited financial resources and need of capacity building of health staffs on NCDs prevention and control is a major limiting factor for implementing NCDs activities throughout the country.—P6

## Lack of supporting policies on NCD prevention and control

Participants also reported that the MSAP was not supported by other NCD policies, suggesting that although policies in the health sectors were related, none were specific to NCDs, with the latest National Health Policy (2014) also lacking in this area.

Our national policies of health sector do not have any specific policies to address NCDs. However, we are formulating [a] new health policy which has specific separate policy provision on NCDs.—P7

## Facilitators to implementation of, and participation in, MSAP

### International practices and commitments

Participants recognized that NCDs were a global health problem, for which a global response is required. They discussed the global and regional attention NCDs had received, including at the UN General Assembly, the WHO Global Action Plan for the prevention and control of NCDs, and the WHO Office for South-East Asia’s (SEARO’s) regional action plan.

Following international practices, we are developing action plans like other health issues and it’s our obligation to implement and achieve the targets we had set.—P7.

Participants reported that Nepal was obligated to prepare the MSAP in 2014 for the period 2014–2020 as it was a WHO Member State and they recognized that it was a responsibility of the Nepal government to implement the planned MSAP activities. They described that Nepal made commitments at national and international levels to control and prevent the NCD burden through effective interventions to reduce risks factors, such as controlling tobacco and alcohol use. With the country being obligated to implement these commitments for the achievement of national targets such as those linked to the SDGs.

We have committed in UN General Assembly as well as World Health Assembly for reducing burden of NCDs. Hence, we should proactively work for prevention and control of NCDs in Nepal with sector collaboration … As per our commitment made in World Health Assembly, we formulated MSAP adopting global and regional action-P7

## Establishment of an NCD section

Participants reported that the establishment of a separate section for NCDs and mental health had facilitated a focus on NCDs in Nepal. Recently, the NCD and Mental Health Section was established under the Epidemiology and Disease Control Division (EDCD) of the Department of Health Services, with terms of reference to facilitate work on NCDs in the country.

In the new organogram of Ministry of Health and Population, NCD and Mental Health Section is established under Epidemiology and Disease Control Division of Department of Health Services and has given TOR (Terms of Reference) to implement activities related to NCDs. This has made us clear to work on NCDs—P9

## Enabling policy environment

The creation of supporting legislation and policy was also seen as important in supporting a focus on NCDs. After formulation of the Constitution of Nepal (2015), basic health services were established as a fundamental right of Nepalese citizens, which was supported through formulation of the Public Health Service Act 2018. Hence, the government is obligated to provide basic health services for NCDs to all citizens free of cost, including the provision of medication through free health service programmes.

Providing treatment to people suffering from diseases such as NCDs is [a] human right after formulation of Constitution of Nepal and enforcement of Public Health Act 2018. Hence, we should work towards NCDs prevention and control in Nepal.—P5

## Health in all policies

Participants reported that inclusion of Health in All policies in the Public Health Service Act 2018 supported the MSAP. They saw this as a policy acknowledgement that the health sector alone could not improve the health status of the population and that responsibilities fell on all sectors. They felt that this improved the accountability of policymakers for health impacts at all levels of policymaking.

Our Public Health Act 2018 has been made considering principles of Health in All policies and this Act has provided broader scope and right to Ministry of Health and Population to prevent and control risk factors of NCDs such as air pollution, noise pollution and chemicals use which adversely affect health of people.—P5

## Resource allocation through the annual work plan and budget

The Government of Nepal allocates budgets through an annual work plan and budget. Commonly called the red book it covers research, surveys, capacity building, advocacy and service delivery programmes. Allocation of resources through this on NCDs prevention and control was recognised as a facilitator in encouraging multi-sectoral participation in the MSAP. Although some suggested that the allocated resources did not reflect the burden of NCDs within the country.

Actually, we have just started including budget for NCDs prevention and control programme in our annual work plan and budget including in [the] red book. But, still allocation of budget is very low compared with the magnitude of NCDs burden in the country.-P7

## Discussion

Findings from this study suggest that stakeholders from sectors involved in the MSAP in Nepal are aware of NCDs as a growing burden and recognise the role that their sectors can play in the prevention and control of them. However, they reported a lack of knowledge around the national MSAP and a low level of engagement. Many felt that institutional reform, occurring after adoption of the MSAP, along with a lack of devolution beyond the national level were reasons for this. Additional barriers to implementation identified by participants included poor leadership, weak dissemination, limited resources and a lack of supporting policies. Participants reported that international commitments to multisectoral work, supported by establishment of a specialist NCD section within the MOHP, along with resource allocation would encourage participation in the MSAP. Also acknowledging the importance of evidence and the promotion of a Health in All policies approach in national policies.

A limitation of this study is that we only obtained data from a certain number of individuals from key sectors, we do not, therefore, provide views from the whole range of stakeholders involved in NCD prevention and control. As our work was carried out in Nepal only, it may have low external validity. However, the sharing of within country experiences has been encouraged and many of the factors identified within the present study are likely to be found in other countries, particularly those that are low income and/or from the South East Asian region [25,26]. Having all the interviews carried out by the same researcher (MD), with experience in qualitative methods, allowed for consistency in data collection. The semi-structured nature of the interviews enabled participants to raise issues relevant to them and allowed an in-depth exploration of the topics raised. A schedule was used to guide the interviews, designed by the research team, in order to limit researcher bias. Two researchers coded the transcripts, with support from the interviewer, to enable researcher triangulation.

At the time of submission, the authors had identified no papers that had used a qualitative approach to study the experiences of individuals from a range of sectors in implementing national multisectoral action plans. A similar study with policy makers from four countries within the WHO Eastern Mediterranean Region (EMR), identified a number of barriers to the development of national MSAPs which were similar to those identified in the present study on implementation [27]. These included a lack of continuity in participation from other sectors, political instability, a lack of understanding of roles of non-health sectors in NCD prevention and control, and NCDs being viewed as a health sector issue. As well as recommending a projected need for further research to identify best practices and challenges in the later implementation stages of the MSAP, which our study aimed to do, participants in the EMR study advised that multisectoral action plans should be considered dynamic and able to respond to political and social change [27]. This links to the political change identified in the current study as a barrier. The WHO SEAR review of MSAPs within the region also identified a lack of adequate human and financial resources, divergent sectoral mandates, industry interference, political pressures and lack of clarity of roles, among other challenges to multisectoral response in the region [24]. Identifying key priority areas for Nepal specifically, which aligned to many of the themes raised by participants in the present study, including equipping the newly constituted provincial and local governments on their roles and responsibilities in the multisectoral response to NCDs, utilizing the opportunity of administrative restructuring to establish subnational coordination mechanisms for NCDs, and developing terms of engagement for non-State actors and the private sector in NCD interventions at the national and subnational levels [24].

‘Time to deliver’, the report of the WHO Independent High-level Commission on Noncommunicable Diseases identified seven overarching barriers to the implementation of multisectoral and multi-stakeholder national coordination mechanisms on NCDs, many of which overlap with the experiences of participants within this study: (1) lack of political will, commitment, capacity, and action; (2) lack of policies and plans for NCDs; (3) difficulty in priority-setting; (4) impact of economic, commercial, and market factors; (5) insufficient technical and operational capacity; (6) insufficient (domestic and international) financing to scale up national NCD responses; and (7) lack of accountability [28].

Previously published guidelines have also identified steps in the development and implementation of NCD MSAPs [3]. In particular, the WHO tool box [29] which provides technical assistance to develop and implement national NCD MSAPs. This was developed from expert opinion rather than country-level experience and suggestions have been made that such guidance is updated as country level experience is built. Similar studies to this one, would therefore be useful both to review implementation within countries and so that other Member States can learn from their experiences.

## Conclusion

Despite strong political calls, implementation of national multisectoral action plans has been found to be challenging. Reflections on the challenges and facilitators to implementation will enable countries to identify common challenges and recommend best practice. Regular opportunities to share national experience will help countries to achieve the target of having an operational NCD MSAP and contribute to the NCD-related SDG targets by 2030. Based on identified barriers and facilitators on this study, multisectoral action plans beyond 2020, should engage stakeholders from federal, provincial and local governments and develop costed action plans with specific roles and responsibilities of each sector. Technical recommendations that arise from the present study include the completion of a wider stakeholder consultation during formulation of MSAP plans; demarking responsibilities of federal, provincial and local governments in NCDs prevention and control; wider circulation and dissemination of the MSAP plan among all Ministries and Departments and finally; arranging regular meetings of multi-sectoral committees to track progress of implementation, including a mid-term evaluation.

## Paper context

WHO Member States agreed to consider developing, or strengthening, national multisectoral policies or plans for the prevention and control of noncommunicable diseases in 2014. However, implementation of these have been challenging, with data suggesting that less than half of reporting countries have an operational national multi-sectoral commission, agency or mechanism. This has led to calls for the development of an implementation research agenda in the governance of multisectoral action. Promoting the exchange of experiences of countries in implementing mechanisms and action plans to support knowledge management and convene peer learning. This paper does that, in documenting the perspectives of key stakeholders on the barriers and facilitators to the implementation of a multisectoral action plan on NCDs in Nepal.
